# Acute Intermittent Porphyria in a Man with Dual Enzyme Deficiencies

**DOI:** 10.1155/2020/8873219

**Published:** 2020-10-15

**Authors:** G. N. Cerbino, L. Abou Assali, L. S. Varela, L. Tomassi, A. Batlle, V. E. Parera, M. V. Rossetti

**Affiliations:** ^1^Centro de Investigaciones sobre Porfirinas y Porfirias (CIPYP)-CONICET, Hospital de Clínicas-UBA, Buenos Aires, Argentina; ^2^Hospital General de Agudos, Buenos Aires, Argentina

## Abstract

Porphyrias are a heterogeneous group of metabolic disorders that result from the altered activity of specific enzymes of the heme biosynthetic pathway and are characterized by accumulation of pathway intermediates. Porphyria cutanea tarda (PCT) is the most common porphyria and is due to deficient activity of uroporphyrinogen decarboxylase (UROD). Acute intermittent porphyria (AIP) is the most common of the acute hepatic porphyrias, caused by decreased activity of hydroxymethylbilane synthase (HMBS). An Argentinean man with a family history of PCT who carried the *UROD* variant c.10_11insA suffered severe abdominal pain. Biochemical testing was consistent with AIP, and molecular analysis of *HMBS* revealed a *de novo* variant: c.344 + 2_ + 5delTAAG. This is one of the few cases of porphyria identified with both *UROD* and *HMBS* mutations and the first confirmed case of porphyria with dual enzyme deficiencies in Argentina.

## 1. Introduction

Porphyria cutanea tarda (PCT), the most common type of porphyria, is due to decreased hepatic activity of uroporphyrinogen decarboxylase (UROD; EC 4.1.1.37), the fifth enzyme in the heme biosynthetic pathway [1]. Accumulation of porphyrins causes skin fragility and blistering on sun-exposed areas, often with hyperpigmentation and hypertrichosis. Three clinically similar forms of PCT are sporadic (S-PCT) or type I, OMIM 176090; familial (F-PCT) or type II, OMIM 176100; and type III PCT. F-PCT is an autosomal dominant disorder with low penetrance in which the UROD activity is reduced in all tissues due to pathogenic variants. F-PCT represents about 20–30% of PCT patients, in whom a *UROD* mutation predisposes to the development of the disease. Most PCT patients have S-PCT, which is not associated with *UROD* variants [2–4]. Type III PCT is rare, with familial occurrence in more than one family member in the absence of *UROD* mutations [3].

Acute intermittent porphyria (AIP), OMIM 176000, is the most common of the acute hepatic porphyrias in most countries [5, 6]. It is an autosomal dominant disorder with incomplete penetrance, caused by the deficient activity of hydroxymethylbilane synthase (HMBS), also known as porphobilinogen deaminase (PBGD EC 4.3.1.8). Acute abdominal pain and various peripheral and central nervous system manifestations are the most frequent symptoms, which can be precipitated by diverse exogenous and endogenous porphyrinogenic factors [2, 7]. Symptomatic as well as most clinical asymptomatic patients with AIP excrete increased levels of *δ*-aminolevulinic acid (ALA), porphobilinogen (PBG), and porphyrins, which can be readily measured in urine. Latent patients have normal ALA, PBG, and porphyrin values, and their diagnosis is based mainly on genetic analysis [6, 8].

## 2. Methods and Results

A 68-year-old male presented with skin fragility, scabs, macules, and dark urine. He was diagnosed as having PCT based on marked elevation in urinary porphyrins, with a predominance of highly carboxylated porphyrins (Table 1). Molecular testing detected a *UROD* pathogenic variant, c.10_11insA, which is the most common one found in PCT patients in Argentina [8]. It should be noted that the symptoms in this man have developed for more than 30 years. A combined treatment with eight phlebotomies (500 ml/each) to diminish iron content and OH chloroquine (100 mg/twice a week) till remission of symptoms was performed in this patient.

In 2015, one of his sons, who was the youngest of 5 siblings, presented at the age of 40 with severe abdominal pain, vomiting, and fever but without cutaneous manifestations. Biochemical tests by previously described methods [9] were consistent with AIP (Table 2).

**Figure 1:**
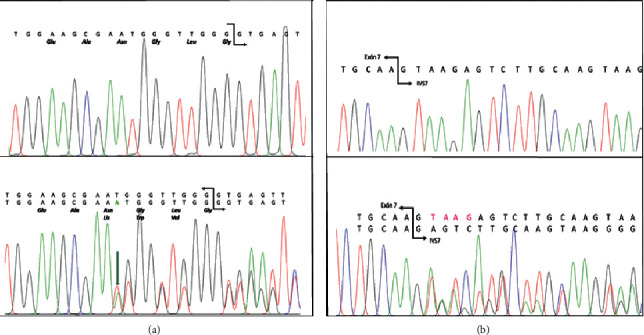
Electropherogram for (a) *UROD* and (b) *HMBS* sequences.

**Figure 2:**
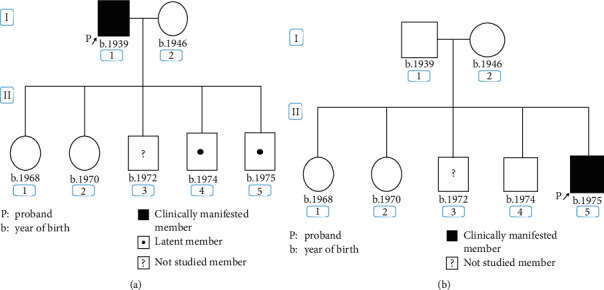
Pedigree of the studied Argentinean family with PCT (a) and AIP (b).

Molecular analysis revealed that he had inherited the *UROD* variant c.10_11insA which was found previously in his father and also harboured a deletion of four bases in intron 7 of *HMBS* (IVS7 + 2_ + 5delTAAG, c.344 + 2_ + 5delTAAG) (Figure 1). This deletion variant was first reported as causing AIP in a patient in Russia [10] and has not been identified previously in Argentina. The patient's parents, two sisters, and one brother were studied (Figure 2).

**Figure 3:**
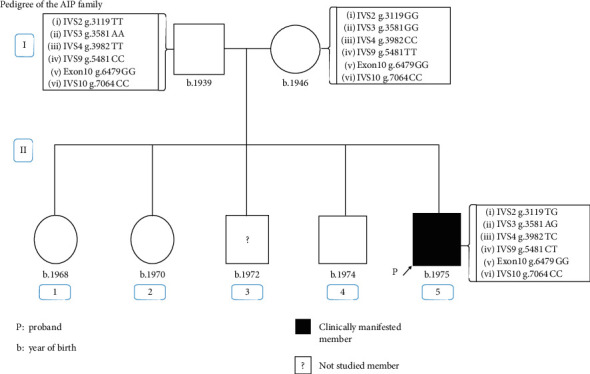
Allele segregation analysis using intragenic SNPs in *HMBS*. SNP underlined indicates a novel SNP in Argentinean population. Position of nucleotide change is numbered relative to the translation initiation codon in Exon 1 in genomic DNA sequence (GenBank accession no. M95623, A in the ATG initiation codon for the housekeeping isoform is +1).

Allele segregation analysis was carried out based on single nucleotide polymorphisms (SNPs) previously studied in AIP families in Argentina. Six intragenic SNPs were found: five of them were identified previously in our population [11], and a novel SNP was found in intron 9 (g.5481C > T) which was inherited from his father, who was of Italian origin (Figure 3).

Management of AIP attacks required treatment and monitoring by the responsible doctor, at J. M. Ramos Mejia Hospital, Attention Center for Porphyric Patients. Oral folic acid (90 mg) was administrated (30 mg/8 h), vitamin B complex (B1, B6 and B12) was consumed orally every 8 hours, and carbohydrate loading (350 g) was the standard treatment for an acute attack of porphyria, following a specific diet. Some symptoms have been treated by propranolol (40 mg/12 h) and clonazepam (0.5 mg/6 h). The treatment also required the psychotherapeutic support. The patient did not work for 3 months till ALA, PBG, and porphyrins values were stabilized. Six months later, the doses of folic acid and B complex were reduced, a suitable diet was maintained, and the patient was stable and without new attacks.

## 3. Discussion

Porphyrias are rare metabolic diseases often misdiagnosed because they share symptoms which are common in many other diseases. Attacks of the acute hepatic porphyrias are triggered by a variety of endogenous and exogenous factors. Early diagnosis is important for advising patients, especially those with latent disease, to avoid such factors and to treat symptoms that do occur promptly to elude severe clinical consequences.

In this family, the father presented with skin lesions at the age of 68 and was found to have biochemical changes typical of PCT and carried c.10_11insA in *UROD*, which is the most frequent variant associated with type II PCT in Argentina [12]. He had 3 sons and 2 daughters, none of whom had developed cutaneous manifestations. In 2015, one son developed severe abdominal pain, a symptom not attributable to PCT. Biochemical finding was consistent with AIP. *UROD* and *HMBS* were analysed, and the results showed the *UROD* familial variant was inherited from his father, but the *HMBS* variant was only found in this patient. The same *HMBS* mutation was previously reported as a novel variant associated with symptomatic AIP in Russia [13].

We carried out genetic analysis in his family (one brother did not consent to be studied). Only one of the analysed siblings carried the familial *UROD* variant.

Allele segregation analysis based on SNPs, already described in our population [14], indicated that the proband's *HMBS* variant was not found in either parent providing evidence for a *de novo* mutation.

Porphyrias are infrequent diseases, but it is even more rare to find mutations in different genes that may cause two types of porphyria in one individual [13, 15–23]. In our studied case, a *UROD* variant was inherited from one parent, and the patient had typical findings of AIP due to a *de novo HMBS* variant. In contrast to his father, he did not develop features of PCT. Whether the inherited *UROD* mutation contributed to the severity of AIP is uncertain, although we found an unusual urinary pattern in this case. Both *UROD* and *HMBS* mutations have already been individually reported. This is the first instance in Argentina where these variants were found together in the same individual, being one of them a *de novo* mutation. It is interesting to note that *de novo* mutations are very rare events in porphyrias [24, 25].

## 4. Conclusions

A *de novo* variant (c.344 + 2_ + 5delTAAG) was identified for the first time in Argentina. This is the first report of a patient with dual porphyria (AIP/PCT) in Argentina with molecular confirmation.

It must be emphasized the importance of carrying out a complete clinical, biochemical, and molecular study to apply the specific treatment, which is critical in cases of coexistence of a cutaneous porphyria with an acute porphyria since the therapy and medical care in both cases are rather different.

## Figures and Tables

**Table 1:** Biochemical analyses in the father.

Date	Qualitative PBG test^*∗*^	ALA, mg/24 h	PBG, mg/24 h	UTP, *µ*g/24 h	PPI (*λ*, 619 nm)	Urinary thin layer chromatography (UTLC, %)				
						Coproporphyrin	Pentaporphyrin	Hexaporphyrin	Heptaporphyrin	Uroporphyrin
18.07.07	Negative			4456	5.8	10	5	5	30	50
21.01.08				1392						
11.09.08				231						
12.03.09				118						
21.12.10				186						
08.02.12				1385						
27.06.12				134						
16.01.13				65						
28.05.14				107						
18.11.15	Negative	1	1.1	160						

Parameters determined: UTP, urinary total porphyrins. PPI, plasma porphyrin index. Normal values: ALA ≤ 4; PBG ≤ 2; UTP 20–250; PPI ≤ 1,30. ^*∗*^Using Ehrlich reactive for PBG detection.

**Table 2:** Biochemical analyses in the son.

Date	Qualitative PBG test	ALA, mg/24 h	PBG, mg/24 h	UTP, *µ*g/24 h	PPI (*λ*, 619 nm)	HMBS, U/ml GR	UTLC (%)
09.10.15	Positive			2316	3.07		Unusual pattern
04.11.15		11.3	38.7	1318	2.17	57.78	
21.12.15		10.9	31.7	1131			
23.03.16		4.7	13.4	1612			
21.07.16		2.5	5.0	991			
01.03.17	Positive	1.5	5.2	591			

Normal value of HMBS: 73,3 ± 13,6.

## Data Availability

The datasets used and/or analysed during the current study are available from the corresponding author on reasonable request.

## Abbreviations

A: Adenine
AIP: Acute intermittent porphyria
ALA: *δ*-Aminolevulinic acid
bp: Base pairs
C: Cytosine
del: Deletion
DNA: Deoxyribonucleic acid
G: Guanine
HMB: Hydroxymethylbilane
HMBS: Hydroxymethylbilane synthase
ins: Insertion
IVS: Intervening sequence
kb: Kilobases
PBG: Porphobilinogen
PBGD: Porphobilinogen deaminase
PCR: Polymerase chain reaction
PCT: Porphyria cutanea tarda
SNP: Single nucleotide polymorphism
T: Thymine
UROD: Uroporphyrinogen decarboxylase.
